# Stroke Lesion Location Is Associated with Dynamic Inspiratory Performance: A Cross-Sectional Study

**DOI:** 10.3390/arm94050076

**Published:** 2026-10-20

**Authors:** Ho-Chieh Kuo, Guanghua Liu, Zongye Zhong, Bangzhong Liu, Xiaokai Zhang, Mingfang Shi

**Affiliations:** 1 Department of Rehabilitation Medicine, Zhongshan Hospital, Fudan University, Shanghai 200032, China; guo.hejie@zs-hospital.sh.cn (H.-C.K.); liu.guanghua@zs-hospital.sh.cn (G.L.); zhong.zongye@zs-hospital.sh.cn (Z.Z.); liu.bangzhong@zs-hospital.sh.cn (B.L.); zhang.xiaokai@zs-hospital.sh.cn (X.Z.); 2 Shanghai Institute of Rehabilitation with Integrated Western and Chinese Traditional Medicine, Shanghai 200032, China

**Keywords:** stroke, lesion location, peak inspiratory flow, respiratory muscles, pulmonary function, rehabilitation

## Abstract

**Highlights:**

**What are the main findings?**

- Across seven respiratory outcomes, peak inspiratory flow (PIF) retained an association with broad lesion-location categories after Holm correction and adjustment for the original six covariates.
- PIF was higher in the basal ganglia region group than in the brainstem group. The association persisted in the full-cohort clinical-adjustment models and ischemic-stroke subgroup, but was weaker in the first-ever-stroke subgroup.

**What are the implications of the main findings?**

- Dynamic inspiratory assessment may help characterize respiratory heterogeneity after stroke, although residual confounding and measurement limitations remain.
- PIF warrants prospective investigation alongside detailed lesion anatomy, stroke severity, functional status, and respiratory physiology.

**Abstract:**

Background/Objectives: We examined whether respiratory performance differed among broad stroke lesion-location categories. Methods: This exploratory single-center cross-sectional analysis combined pre-intervention assessments from 20 participants in an earlier trial with 60 subsequently recruited participants. The 80 patients were classified into frontal lobe (*n* = 27), basal ganglia region (*n* = 26), or brainstem (*n* = 27) categories. Peak inspiratory flow (PIF), strength index (S-Index), and five conventional respiratory outcomes were assessed. Holm correction was applied across seven outcomes. Linear models used type 3 heteroscedasticity-consistent (HC3) robust standard errors and the original six clinical covariates. Additional PIF analyses incorporated time since stroke, National Institutes of Health Stroke Scale (NIHSS), or modified Barthel Index (MBI), and restricted the sample to ischemic or first-ever stroke. Results: Only PIF retained an unadjusted group association after Holm correction (adjusted *p* = 0.008). The adjusted overall lesion-location association also survived correction (adjusted *p* = 0.009). PIF was 0.625 L/s higher in the basal ganglia region than in the brainstem group (95% confidence interval [CI], 0.282–0.967). This contrast persisted with additional clinical adjustment in the full cohort and in ischemic stroke. Evidence weakened in first-ever stroke; with MBI adjustment, the contrast was 0.381 L/s (95% CI, −0.067–0.829). Conclusions: Broad lesion-location categories were associated with PIF in this selected cohort. The findings remain exploratory and cannot fully separate anatomical associations from stroke severity, disability, unmeasured clinical factors, or measurement limitations.

## 1. Introduction

Respiratory impairment is an underrecognized component of post-stroke disability. Stroke survivors can exhibit reduced respiratory muscle strength, impaired pulmonary function, and altered diaphragmatic mechanics, and these abnormalities may coexist with motor and functional deficits [1,2,3,4]. Yet stroke is often treated as a single entity in respiratory studies, leaving the neuroanatomical heterogeneity of respiratory performance comparatively underexplored.

Respiratory control is distributed across automatic brainstem circuits and supratentorial networks involved in voluntary respiratory behavior. Neurophysiological studies have demonstrated disruption of corticodiaphragmatic pathways after stroke, while functional neuroimaging and intracranial recordings implicate frontal, basal ganglia, thalamic, sensorimotor, and pontine regions during voluntary breathing, breath holding, and brisk sniffing [5,6,7,8]. Lesion-based clinical studies have also linked stroke location to voluntary cough performance, including lesion-type differences and specific supratentorial regions associated with weak cough flow [9,10].

These observations raise a more specific question: does lesion-related heterogeneity extend to rapid dynamic inspiration? Peak inspiratory flow (PIF) captures the maximal airflow generated during a rapid, forceful inspiratory maneuver and can be measured reliably with an electronic inspiratory loading device in post-stroke patients [11]. The device-derived strength index (S-Index) reflects dynamically generated inspiratory pressure and is not equivalent to conventional static maximal inspiratory pressure; it is also strongly flow-dependent [12,13,14].

Accordingly, we compared PIF, S-Index, and conventional pulmonary function indices among patients categorized as having frontal lobe, basal ganglia region, or brainstem lesions. We examined these associations after adjustment for the original clinical covariates and in additional analyses addressing time since stroke, neurological severity, functional status, and stroke history. The analysis was exploratory and intended to characterize associations rather than infer causal effects of lesion location.

## 2. Materials and Methods

### 2.1. Study Design and Participants

This exploratory single-center cross-sectional analysis combined baseline respiratory assessments from two recruitment periods at the Department of Rehabilitation Medicine, Zhongshan Hospital, Fudan University, Shanghai, China. The earlier source was a 30-participant respiratory-training trial recruited between November 2022 and August 2023 [15]. These 30 enrolled trial participants were assessed for inclusion in the present analysis, of whom 20 were included. During subsequent consecutive screening between November 2024 and January 2026, 90 patients were assessed and 60 were included. Thus, 120 individuals were assessed for inclusion in this analysis, yielding an analytical sample of 80. The 30 earlier participants do not represent the source trial’s original screening denominator. Only pre-intervention measurements were used; this analysis did not evaluate training effects, and anatomical categories did not represent randomized treatment allocation.

**Figure 1:**
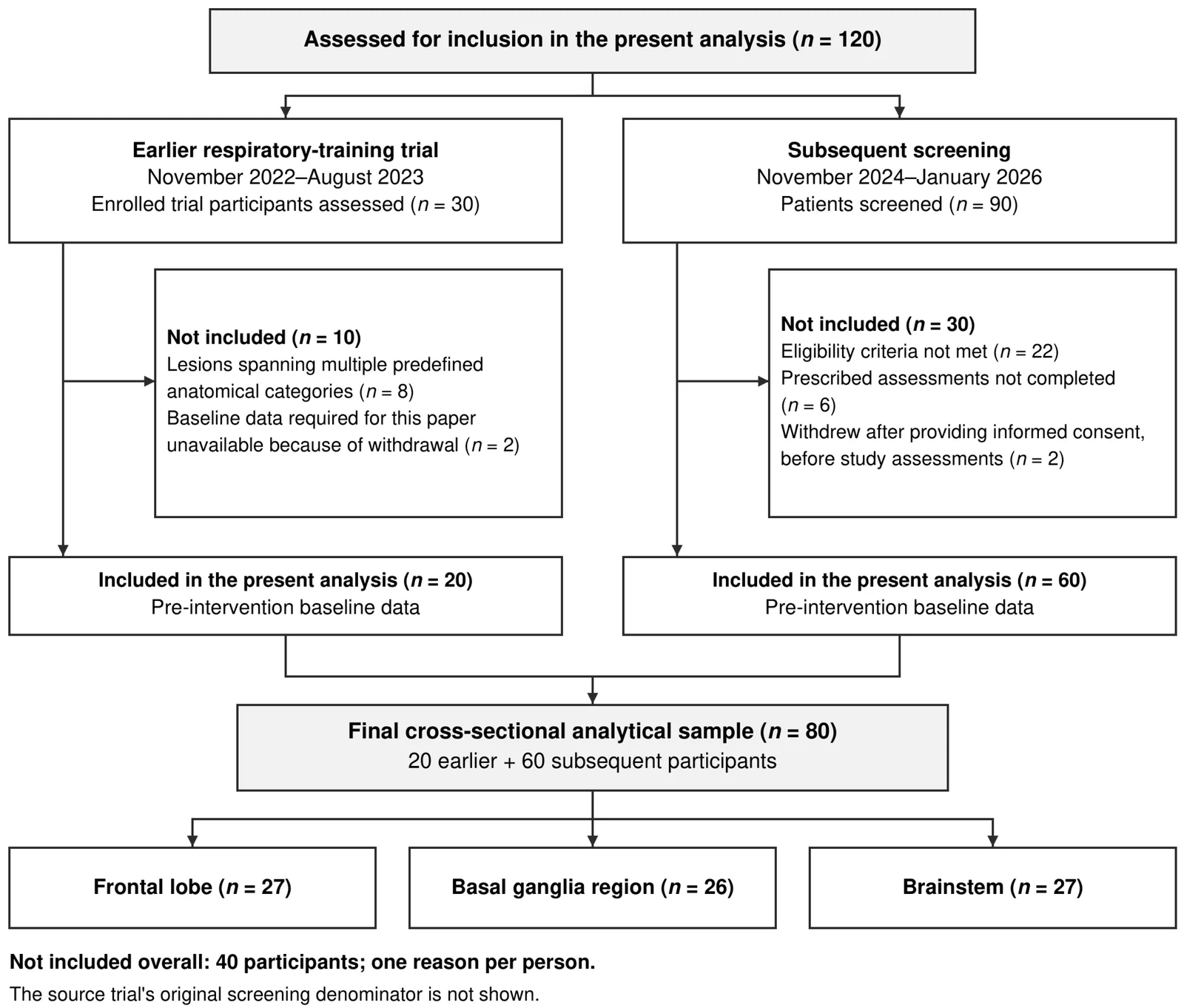
Two-stage participant selection for the exploratory cross-sectional analysis. Of 120 individuals assessed for inclusion, 30 were enrolled participants from the earlier respiratory-training trial and 90 were subsequently screened patients. Stage-specific reasons for non-inclusion are shown for 10 earlier and 30 subsequent individuals, with one reason counted per person. The earlier trial’s original screening denominator is not shown. Lesion-based non-inclusion in this analysis does not indicate withdrawal from the source trial. Two patients withdrew consent after providing informed consent but before study assessments. The final 80 participants comprised frontal lobe (*n* = 27), basal ganglia region (*n* = 26), and brainstem (*n* = 27) categories; these were anatomical groups, not randomized treatment arms.

Reasons for non-inclusion were reported separately by recruitment period, with each person counted under one reason. In the earlier trial cohort, eight participants had lesions spanning multiple predefined anatomical categories, and two lacked baseline data required for this paper because of withdrawal. The eight lesion-based exclusions concern the present analysis and do not indicate withdrawal from the source trial. In the subsequent screening period, 22 patients did not meet eligibility criteria, six did not complete the prescribed assessments, and two withdrew consent after providing informed consent but before study assessments. The latter two were not classified as lost to follow-up. The two periods contributed 10 and 30 non-included individuals, respectively (Figure 1).

The present analysis applied its own eligibility criteria to both sources. Eligible patients were aged 20–90 years, had clinically confirmed ischemic or hemorrhagic stroke with cranial imaging, had a Mini-Mental State Examination score ≥24, and could understand and complete respiratory testing. Patients with cognitive impairment, facial motor impairment, dysphagia, an inadequate mouthpiece seal, or clinically critical or unstable illness were excluded. The exclusion of critical or unstable illness was a clinical criterion, not a numerical NIHSS severity cutoff. Other exclusions were a history of thoracic or abdominal surgery, another neurological disorder likely to substantially affect respiratory muscle function, congenital thoracic deformity or rib fracture, and lesions involving more than one predefined study category. These criteria also applied to the 20 earlier participants.

Patients with known chronic obstructive pulmonary disease (COPD), asthma, restrictive pulmonary disease, heart failure, recent pneumonia, or a history of mechanical ventilation were excluded. These clinical exclusions did not constitute systematic quantification of disease severity or exclude undiagnosed cardiopulmonary disease. Coronary heart disease was recorded separately and was not an exclusion. Baseline assessments preceded the study-related respiratory rehabilitation intervention; they do not establish absence of all previous rehabilitation exposure.

Subsequent recruitment was consecutive, and respiratory testing followed a prospectively implemented baseline assessment procedure. The National Institutes of Health Stroke Scale (NIHSS) and the modified Barthel Index (MBI; 0–100 points) were recorded on admission, and respiratory testing was performed the following day. Time since stroke was defined as the interval in days between the index stroke onset and respiratory testing. No longitudinal respiratory outcome or training response was analyzed in this paper.

All participants provided written informed consent. The research project received approval from the Ethics Committee of Zhongshan Hospital, Fudan University (approval No. B2022-490R; approved on 30 September 2022). The earlier respiratory-training trial was registered in the Chinese Clinical Trial Registry (ChiCTR2300067871; 30 January 2023); the present cross-sectional analysis was not independently registered. Reporting follows the Strengthening the Reporting of Observational Studies in Epidemiology (STROBE) statement [16].

### 2.2. Lesion Assessment and Classification

All 80 participants had both cranial computed tomography (CT) and magnetic resonance imaging (MRI) available. Two physicians experienced in stroke imaging independently reviewed the images while blinded to PIF, S-Index, and pulmonary function results. Three predefined study categories were used: frontal lobe, basal ganglia region, and brainstem. The frontal category included frontal cortex and subcortical white matter. The basal ganglia region category required involvement of basal ganglia structures such as the caudate nucleus, putamen, or globus pallidus and allowed accompanying involvement of the adjacent internal capsule. It was therefore a regional category, not a nucleus-specific category. No participant in this sample had thalamic involvement. The brainstem category included midbrain, pontine, and medullary lesions.

Eligibility required involvement of a single predefined study category. This did not require a lesion to affect only one structure within that category. Lesions involving two or more predefined categories were excluded. Classification was based on the index stroke, with old lesions also considered for eligibility; in included participants with previous stroke, old and index lesions were within the same predefined category. Disagreements were resolved by discussion, with persistent disagreement adjudicated by a third senior physician. Respiratory results were not used to revise lesion classification.

Initial inter-rater agreement was calculated from the two physicians’ independent classifications before consensus. We report the disagreement proportion and unweighted Cohen’s κ with a 95% confidence interval (CI) calculated using the asymptotic standard error. Laterality and brainstem level were available, but lesion volume, standardized segmentation, and complete structure-level involvement counts were not quantified.

### 2.3. Dynamic Inspiratory Assessment

Demographic and clinical data included age, sex, height, weight, body mass index (BMI), smoking history, hypertension, coronary heart disease, previous stroke, stroke type, and side of hemiplegia. Smoking pack-years were recorded for the 42 participants with a smoking history. Blank pack-year entries for the 38 participants with a documented absence of smoking history denoted zero cumulative exposure and were coded as 0; these zeros did not represent imputation of unknown smoking exposure. Pack-years were therefore available for description and supplementary analysis of the full cohort.

All respiratory assessments were performed by one respiratory therapist who did not know the patients’ lesion categories before testing. The same procedure, instructions, and encouragement were used for all participants. Dynamic inspiratory performance was measured using POWERbreathe K5 (POWERbreathe International Ltd., Southam, UK), following the dynamic open-airway S-Index assessment described by Areias et al. [17]. The device was operated in Test mode without an added inspiratory training load. Equipment preparation followed the manufacturer’s operating instructions. Participants sat upright, wore a nose clip, maintained a mouthpiece seal, exhaled fully, and then inhaled as rapidly, forcefully, and deeply as possible. A technically acceptable maneuver required a maintained seal and completion of the instructed inspiratory effort. The device was returned to the manufacturer annually for recalibration and maintenance. This practice was confirmed by the investigators; manufacturer service certificates and detailed calibration logs were unavailable for retrospective verification.

Two familiarization maneuvers preceded three formal inspiratory efforts, with 30 s rest intervals. The procedural repeatability criterion was a difference < 10% across three S-Index efforts, based on Areias et al. [17]. PIF (L/s) and S-Index (cmH_2_O) were recorded from the same efforts, and the arithmetic mean of the three formal measurements was used to summarize within-session dynamic performance consistently across participants. This averaging choice was specific to the present analysis and was not attributed to the reference protocol. S-Index was interpreted as a dynamic device-derived index, not conventional static maximal inspiratory pressure [11,12,13,14,17]. During subsequent screening, six patients did not complete the prescribed assessments and were not included in the analysis (Figure 1).

The retained analytical dataset contains session-level summaries rather than individual-effort recordings. It does not permit retrospective calculation of cohort-specific repeatability rates or reconstruction of the handling of formal efforts that did not meet the repeatability criterion. Meeting the procedural S-Index criterion was not taken as evidence that PIF independently satisfied the same threshold.

### 2.4. Pulmonary Function Assessment

Pulmonary function was measured with an AS-507 system (Minato Medical Science Co., Ltd., Osaka, Japan), with participants seated. Vital capacity (VC), forced vital capacity (FVC), forced expiratory volume in one second (FEV1), peak expiratory flow (PEF), and maximum voluntary ventilation (MVV) were recorded. Spirometric testing and final FEV1 and FVC value selection followed the applicable American Thoracic Society (ATS)/European Respiratory Society (ERS) technical standards; the final values were not arithmetic means of three maneuvers [18,19]. Five participant records contained transposed FVC and FEV1 entries (five pairs, corresponding to ten measurement cells). The corrections were verified against the investigators’ case-specific confirmation and the affected spreadsheet rows and original values. The original spirometry curves were unavailable for independent retrospective verification. Corrected values were used to recompute FVC and FEV1 descriptive statistics, unadjusted group comparisons, adjusted outcome models and relevant model diagnostics, and participant-level FEV1/FVC ratios and group comparisons. Both seven-outcome Holm families were recalculated from unrounded omnibus *p* values.

FEV1/FVC was calculated for each participant from the retained final FEV1 and FVC values and expressed as a percentage. Original percent-predicted spirometry records were lost. Raw curves, individual spirometric efforts, and quality grades were not available for retrospective verification in this revision. Thus, we cannot report the proportion meeting acceptability and repeatability criteria, assign retrospective quality grades, or quantify the number of rejected or repeated maneuvers. Adherence to the testing procedure is distinguished from documented cohort-specific quality assurance.

### 2.5. Statistical Analysis

Analyses were performed using SPSS version 26.0 and Python version 3.14. Approximately normally distributed continuous variables are reported as mean ± standard deviation (SD); time since stroke, NIHSS, MBI, and smoking pack-years are reported as median (first quartile [Q1], third quartile [Q3]). Categorical variables are reported as *n* (%). Baseline comparisons used one-way analysis of variance (ANOVA), Kruskal–Wallis tests for the added median-based variables, or chi-square tests, as appropriate. Pack-years were compared across lesion categories in the full cohort, including zero exposure in participants without a smoking history; the smoking-history subset was described separately. Respiratory outcomes with equal variances were compared using one-way ANOVA and Tukey post hoc tests; unequal-variance outcomes used Welch ANOVA and Games–Howell comparisons. FEV1/FVC was a supplementary outcome, compared using ANOVA with an additional Kruskal–Wallis check.

The original outcome family comprised PIF, S-Index, VC, FVC, FEV1, PEF, and MVV. Holm correction was applied separately to the seven unadjusted omnibus group tests and the seven adjusted omnibus lesion-location tests, using unrounded *p* values before final reporting. Supplementary Table S7 provides the numerical audit. Separate ordinary least-squares models used brainstem as the reference category and adjusted for age, sex, height, smoking history, coronary heart disease, and stroke type. The six-item adjustment set was defined during the original project planning on clinical grounds, before registration or recruitment, and was not selected according to baseline between-group *p* values or respiratory outcome significance. Age, sex, and height account for major demographic influences on respiratory measurements; smoking history represents recorded respiratory exposure; coronary heart disease represents a recorded cardiovascular comorbidity; and stroke type addresses ischemic–hemorrhagic heterogeneity. This rationale does not imply that the adjustment set captures all confounding or that it was registered for the current 80-participant analysis. BMI and previous stroke were recorded but were not included in the original adjustment set.

Type 3 heteroscedasticity-consistent (HC3) robust standard errors were used [20]. Coefficient CIs and *p* values used a normal approximation, and the joint lesion-location test used a two-degree-of-freedom HC3 Wald χ^2^ statistic. Supplementary PIF models added time since stroke, followed by NIHSS or MBI in separate models. NIHSS and MBI were not entered together. The original and supplementary clinical-adjustment PIF models were repeated in ischemic stroke and first-ever stroke; stroke type was omitted from ischemic-only models. A further PIF analysis was restricted to the 42 participants with a smoking history and complete pack-year data. It retained age, sex, height, coronary heart disease, and stroke type and compared estimates before and after adding continuous pack-years; the smoking-history indicator was omitted because it was constant in this subset. A separate full-cohort PIF sensitivity analysis replaced binary smoking history with continuous pack-years, retaining the other original covariates. The zero-exposure definition for the 38 participants without a smoking history was applied in this model. Binary smoking history and continuous pack-years were not entered together in this replacement model. These revision-stage analyses and the FEV1/FVC comparison were exploratory, with nominal *p* values, and did not expand or replace the original seven-outcome Holm family. Because PIF and S-Index derive from the same efforts, they were not treated as independent confirmatory endpoints.

Model assessment in the full and ischemic cohorts included variance inflation factors (VIFs), residual-versus-fitted and Q–Q plots, partial residual plots, Shapiro–Wilk and Breusch–Pagan screens, and an HC3 Wald screen for added squared and cubed fitted-value terms. Influence assessment used Cook’s distance, leverage, externally studentized residuals, and DFBETAS. Thresholds of Cook’s D > 4/*n*, leverage > 2k/*n*, |studentized residual| > 3, and |DFBETAS| > 2/√*n* were screening guides, where k includes the intercept [21]. Additional PIF checks refitted each model after deleting one participant at a time and after jointly omitting Cook-flagged observations; the primary models retained all participants. Adjusted R^2^ and lesion-category partial R^2^ are reported, the latter representing the fraction of residual variation explained by adding the two lesion terms to the covariate-only model. Diagnostic *p* values were exploratory, not proof that assumptions were satisfied.

All tests were two-sided. Statistical significance for the original outcome family was assessed using Holm-adjusted *p* values; other analyses used nominal *p* values with emphasis on effect estimates and CIs. No a priori sample-size calculation for a lesion-location effect was performed. No values were imputed for the analyzed respiratory outcomes, the original covariates, time since stroke, NIHSS, or MBI.

## 3. Results

Of 120 individuals assessed for inclusion in the present analysis, 80 were analyzed and 40 were not included. Among the 30 enrolled participants from the earlier trial, 20 were included; eight were excluded because lesions involved multiple predefined anatomical categories, and two lacked baseline data required for this paper because of withdrawal. Among the 90 subsequently screened patients, 60 were included; 22 did not meet eligibility criteria, six did not complete the prescribed assessments, and two withdrew consent after providing informed consent but before study assessments. Each of the 40 non-included individuals was counted under one reason. The eight earlier lesion-based exclusions do not indicate withdrawal from the source trial, and the two later consent withdrawals occurred before study assessments rather than during longitudinal follow-up. The final sample combined 20 earlier and 60 subsequent participants (Figure 1). No longitudinal respiratory outcome was analyzed.

The 80 included participants comprised frontal lobe (*n* = 27), basal ganglia region (*n* = 26), and brainstem (*n* = 27) groups. Age ranged from 32 to 88 years; 55 participants (68.8%) were men, 59 (73.8%) had ischemic stroke, and 63 (78.8%) had first-ever stroke. Time since stroke was 75 (65, 85.25) days overall, with a range of 29–100 days; group medians were 72, 77, and 72 days, respectively (Table 1). Overall NIHSS was 4 (3, 7), and MBI was 55 (40, 71.25). The original demographic and clinical variables showed no statistically significant group differences. In the full cohort, median pack-years were 5 (0, 8) overall and 6 (0, 9), 0 (0, 8), and 5 (0, 8) in the frontal lobe, basal ganglia region, and brainstem groups, respectively (nominal *p* = 0.410). Among the 42 participants with a smoking history, the corresponding group medians were 8 (6, 12), 8 (7.5, 9), and 8 (7, 10) (nominal *p* = 0.993). These descriptive comparisons do not establish equivalence or absence of confounding.

The brainstem group included 18 pontine, 7 midbrain, and 2 medullary lesions. Overall lesion laterality was left in 45 participants and right in 35; this is distinct from the hemiplegia side reported in Table 1. Initial imaging classifications agreed in 74/80 participants (92.5%) and differed in 6/80 (7.5%). Unweighted Cohen’s κ was 0.888 (95% CI, 0.801–0.974; Supplementary Table S1). No values were missing for the variables used in the principal or supplementary clinical-adjustment models.

Unadjusted analyses identified omnibus differences in PIF (*p* = 0.00118), S-Index (*p* = 0.025), and FEV1 (*p* = 0.036). Only PIF retained a significant omnibus association after Holm correction across the seven outcomes (adjusted *p* = 0.008; Table 2). Tukey comparisons showed higher PIF in the basal ganglia region group than in the brainstem group (mean difference, 0.786 L/s; 95% CI, 0.288–1.284; *p* < 0.001) or the frontal group (0.527 L/s; 95% CI, 0.029–1.025; *p* = 0.035); the frontal–brainstem comparison was not significant (*p* = 0.426). These pairwise *p* values are within-outcome post hoc results and are not separately Holm-corrected across all outcomes. The nominal S-Index and FEV1 omnibus findings did not survive outcome-level correction.

With the original six covariates, lesion category remained associated with PIF (overall *p* = 0.00126; Table 3), including after Holm correction (adjusted *p* = 0.009). PIF was 0.625 L/s higher in the basal ganglia region than in the brainstem group (95% CI, 0.282–0.967; *p* < 0.001). The frontal–brainstem estimate was 0.260 L/s (95% CI, −0.031–0.552; *p* = 0.080). Neither S-Index nor FEV1 retained a significant overall association after correction (adjusted *p* = 0.322 and 0.263, respectively), despite nominal basal ganglia region–brainstem contrasts. No conventional respiratory outcome retained a significant adjusted omnibus association after multiplicity correction.

The original ischemic-stroke analysis included 59 participants (Table 4). The adjusted PIF contrast remained positive (basal ganglia region versus brainstem: 0.792 L/s; 95% CI, 0.400–1.185; overall nominal *p* < 0.001). S-Index showed a nominal association in this subgroup (overall *p* = 0.018), whereas the FEV1 association was not reproduced (overall *p* = 0.155).

In the full cohort, the basal ganglia region–brainstem PIF estimates were 0.614 L/s after adding time since stroke (95% CI, 0.269–0.959), 0.636 L/s after additionally adjusting for NIHSS (95% CI, 0.300–0.973), and 0.541 L/s after additionally adjusting for MBI (95% CI, 0.200–0.882). Corresponding estimates in ischemic stroke were 0.791, 0.721, and 0.650 L/s, with CIs excluding zero (Table 5). The MBI-adjusted full-cohort estimate was approximately 13.4% smaller than the original estimate.

In the 63 participants with first-ever stroke, the original-model basal ganglia region–brainstem contrast was 0.507 L/s (95% CI, 0.064–0.950), but the overall three-category test was not significant (*p* = 0.073). Overall tests also remained nonsignificant in the supplementary models. With time since stroke and MBI, the contrast was 0.381 L/s (95% CI, −0.067–0.829; overall *p* = 0.197). Thus, the analyses did not uniformly reproduce the original finding.

FEV1/FVC was 69.95 ± 20.06% overall. Group means were 68.89 ± 19.01%, 74.73 ± 21.46%, and 66.41 ± 19.52% for frontal, basal ganglia region, and brainstem categories, respectively. Neither the supplementary ANOVA (*p* = 0.306) nor Kruskal–Wallis check (*p* = 0.231) identified evidence of a group difference. This does not establish pulmonary equivalence or exclude pulmonary confounding.

For the original PIF models, adjusted R^2^ was 0.463 in the full cohort and 0.415 in ischemic stroke, maximum VIF was 2.01 and 1.90, and lesion-category partial R^2^ was 0.161 and 0.226, respectively (Supplementary Table S2). PIF residual plots and screening tests did not indicate a clear major departure from the specified linear form. Cook’s D > 4/*n* flagged 3 full-cohort and 2 ischemic-stroke observations. Leave-one-out PIF estimates remained positive, with CIs excluding zero for the basal ganglia region–brainstem contrast. Joint omission of flagged observations yielded 0.551 L/s (95% CI, 0.232–0.869) and 0.667 L/s (95% CI, 0.303–1.032), respectively. Some frontal–brainstem contrasts changed their nominal significance in omission analyses; these were not promoted to main findings (Supplementary Table S4).

Diagnostics of secondary-outcome models were less reassuring. PEF and MVV residual normality screens indicated departures in both cohorts, and fitted-value power screens raised nominal concerns for full-cohort S-Index, FVC, FEV1, and MVV and ischemic-stroke FVC (Supplementary Table S3). These exploratory screens do not establish a specific model error, but HC3 standard errors do not resolve functional-form concerns. Supplementary Figures S1 and S2 show the original PIF model diagnostics.

In a revision-stage exploratory sensitivity analysis of all 80 participants, continuous pack-years replaced binary smoking history while age, sex, height, coronary heart disease, and stroke type were retained. The basal ganglia region–brainstem PIF contrast was 0.624 L/s (95% CI, 0.281–0.966; overall nominal *p* = 0.00117), compared with 0.625 L/s (95% CI, 0.282–0.967) in the original model (Supplementary Table S6). Adjusted R^2^ was 0.467 and maximum VIF was 1.99. Leave-one-out overall *p* values ranged from 0.00034 to 0.00253, and the contrast CIs remained above zero. These limited checks do not establish that all model assumptions were satisfied. In the separate revision-stage exploratory subset analysis of 42 participants with a smoking history, additional pack-year adjustment yielded 0.754 L/s (95% CI, 0.200–1.308; overall *p* = 0.028; Supplementary Table S5). Neither analysis replaced the original primary model or its multiplicity correction.

## 4. Discussion

PIF showed the most consistent association with broad lesion-location categories among the seven original outcomes. Its omnibus association survived Holm correction in the unadjusted and original adjusted analyses. The basal ganglia region–brainstem contrast persisted with additional time-since-stroke, NIHSS, or MBI adjustment in the full cohort and in ischemic stroke. However, evidence weakened when analysis was restricted to first-ever stroke. These findings support an exploratory association, not a lesion-location effect demonstrated uniformly across all analyses.

These findings extend prior lesion-respiratory research rather than simply reiterating that stroke impairs breathing. Earlier work demonstrated central corticodiaphragmatic dysfunction after ischemic stroke and lesion-type differences in voluntary cough, while voxel-based lesion-symptom mapping linked weak voluntary cough to frontal and related white-matter lesions [5,9,10]. Our study addresses a different respiratory phenotype: rapid inspiration. This distinction matters because cough is primarily an airway-protective behavior with a major expiratory component, whereas PIF reflects the ability to initiate and generate rapid inspiratory airflow.

The prominence of PIF is physiologically plausible. Rapid inspiratory performance depends on coordinated neural drive, fast inspiratory muscle recruitment, respiratory mechanics, and available lung volume. Functional imaging during voluntary hyperpnea, breath holding, and brisk sniffing demonstrates distributed cortical–subcortical–brainstem recruitment rather than isolated brainstem control [6,7,8]. In people after stroke, diaphragm and accessory inspiratory muscle recruitment also changes as inspiratory load increases [22]. These observations provide a rationale for studying dynamic inspiration, but our data do not establish that PIF is more neurologically sensitive than spirometry because diagnostic sensitivity, responsiveness, and neural drive were not directly measured.

The strongest original adjusted contrast was higher PIF in the basal ganglia region group than in the brainstem group. The original frontal–brainstem contrast was not significant, which does not support a general supratentorial-versus-brainstem distinction. Voluntary breathing recruits distributed networks [6,7,8], but broad regional labels cannot identify a specific nucleus, pathway, or brainstem respiratory center. In particular, accompanying internal-capsule involvement in the basal ganglia region category prevents attribution of the contrast solely to basal ganglia nuclei.

Available clinical adjustment reduced some uncertainty but did not isolate an anatomical mechanism. The basal ganglia region–brainstem estimate was smaller after MBI adjustment, and its CI crossed zero in the first-ever-stroke MBI model. Restricted-sample estimates also had wider CIs. Severity and function can reflect consequences of the stroke as well as clinical heterogeneity; their inclusion is therefore a sensitivity analysis rather than identification of a causal direct effect. Similarly, adjustment for time since stroke does not establish consistency outside the observed 29–100-day range.

S-Index is physiologically related to PIF but does not independently confirm its association. Both originate from the same rapid inspiratory effort, and S-Index is distinct from static maximal inspiratory pressure [12,13,14,17]. In the full cohort, the overall S-Index association was not significant before adjusted multiplicity correction and did not survive that correction. Its nominal basal ganglia region–brainstem contrast and ischemic-stroke results should remain exploratory. FEV1 was also inconsistent: its nominal full-cohort association did not survive Holm correction and was not reproduced in ischemic stroke.

Conventional pulmonary function integrates influences beyond lesion location, including neurological impairment, respiratory muscle function, and trunk control [1,2,3,4,23]. Low absolute FVC, FEV1, PEF, or MVV cannot be attributed wholly to the stroke or used alone to classify obstructive or restrictive disease. The supplementary FEV1/FVC results add context, but the lack of original percent-predicted values, reference-standardized results, and retrospectively verifiable maneuver quality limits clinical interpretation [18,19]. Adjustment for age, sex, and height in association models does not replace reference-standardized clinical interpretation.

PIF model checks suggest that the main contrast was not driven by a single participant, although some observations were influential and some frontal contrasts changed in omission checks. Partial R^2^ describes conditional statistical explanatory contribution, not the causal proportion of respiratory variation attributable to lesion location. Diagnostic concerns for secondary outcomes further limit interpretation of their nominal associations and null results. The findings do not justify lesion-specific respiratory prescriptions or screening thresholds.

Strengths include independent blinded imaging review with quantified inter-rater agreement, respiratory testing by one therapist unaware of lesion category before testing, assessment of dynamic and conventional respiratory measures, HC3 standard errors, and multiplicity correction for the original outcome family. Additional clinical-adjustment and subgroup analyses provide a more qualified account of the PIF association.

The single-center cross-sectional design precludes causal inference, and the modest sample limits precision, especially in restricted samples. Combining recruitment periods may introduce selection bias. Lesion volume, detailed structure-level involvement, and tract integrity were not quantified. The broad categories, allowance of adjacent internal-capsule involvement, and exclusion of lesions involving two or more predefined study categories limit precise neuroanatomical interpretation and generalizability.

Although NIHSS, MBI, and time since stroke were available, the modified Rankin Scale (mRS) and trunk control were not measured. BMI was recorded but not adjusted for in the original model; prior stroke was addressed by restriction, which weakened the evidence. The original model was adjusted for binary smoking history. The revision-stage full-cohort pack-year sensitivity analysis yielded a similar PIF contrast, but this does not eliminate exposure measurement error, model misspecification, or smoking-related residual confounding. Disease-specific severity, acute treatment details, and the full intensity and duration of previous rehabilitation were not systematically quantified. Exclusion of known pulmonary conditions does not rule out undiagnosed disease. Residual confounding remains and may contribute to the PIF differences.

Testing was effort-dependent, and original percent-predicted spirometry records were lost. Unavailable curves, maneuver-level quality records, and detailed K5 equipment and attempt logs limit retrospective verification of measurement quality and repeatability. Patients with cognitive or facial motor impairment, dysphagia, clinically critical or unstable illness, or inability to maintain a seal or complete testing were excluded. The findings may therefore not generalize to these patients. Finally, PIF and S-Index are related outputs of one maneuver, and supplementary analyses were exploratory rather than an independent confirmatory family.

## 5. Conclusions

In this selected cross-sectional cohort, broad lesion-location categories were associated with PIF. The basal ganglia region–brainstem difference persisted after original covariate adjustment and omnibus multiplicity correction, and in full-cohort supplementary clinical-adjustment and ischemic-stroke analyses. Evidence weakened in first-ever stroke, including a CI crossing zero after MBI adjustment. Residual confounding, broad anatomical grouping, and measurement limitations prevent interpretation as an independent anatomical determinant of respiratory performance. Prospective studies with detailed lesion characterization, standardized quality records, and comprehensive clinical assessment are needed.

## Figures and Tables

**Table 1:** Baseline characteristics by lesion-location group.

Characteristic	Overall (*n* = 80)	Frontal Lobe (*n* = 27)	Basal Ganglia Region (*n* = 26)	Brainstem (*n* = 27)	*p* Value
Age, years	70.58 ± 11.45	73.11 ± 7.46	67.50 ± 14.48	71.00 ± 11.17	0.200
Height, m	1.67 ± 0.09	1.67 ± 0.08	1.67 ± 0.09	1.67 ± 0.10	0.982
Weight, kg	68.30 ± 11.10	67.11 ± 12.71	68.73 ± 9.36	69.07 ± 11.22	0.791
BMI, kg/m^2^	24.44 ± 3.54	24.01 ± 4.52	24.69 ± 3.13	24.64 ± 2.82	0.742
Male, *n* (%)	55 (68.8)	20 (74.1)	16 (61.5)	19 (70.4)	0.601
Smoking history, *n* (%)	42 (52.5)	17 (63.0)	11 (42.3)	14 (51.9)	0.321
Hypertension, *n* (%)	67 (83.8)	21 (77.8)	22 (84.6)	24 (88.9)	0.536
Coronary heart disease, *n* (%)	52 (65.0)	17 (63.0)	17 (65.4)	18 (66.7)	0.959
First-ever stroke, *n* (%)	63 (78.8)	23 (85.2)	22 (84.6)	18 (66.7)	0.169
Ischemic stroke, *n* (%)	59 (73.8)	20 (74.1)	21 (80.8)	18 (66.7)	0.506
Right hemiplegia, *n* (%)	38 (47.5)	13 (48.1)	11 (42.3)	14 (51.9)	0.782
Time since stroke, days †	75 (65, 85.25)	72 (64.5, 82)	77 (68.25, 86.75)	72 (62.5, 83)	0.234
NIHSS, points †	4 (3, 7)	4 (2, 6.5)	4 (2, 6)	4 (3.5, 7)	0.635
MBI, points †	55 (40, 71.25)	55 (45, 67.5)	65 (45, 78.75)	55 (35, 65)	0.150
Smoking pack-years full cohort †	5 (0, 8)	6 (0, 9)	0 (0, 8)	5 (0, 8)	0.410
Smoking pack-years among smokers †	8 (7, 10)	8 (6, 12)	8 (7.5, 9)	8 (7, 10)	0.993

Continuous variables are mean ± SD unless marked †, which indicates median (Q1, Q3). Categorical variables are *n* (%). † comparisons use Kruskal–Wallis tests. Full-cohort pack-years include 0 for the 38 participants with documented absence of smoking history; these values are not imputed missing exposures. The smoking-history subset comprises 42 participants (frontal *n* = 17, basal ganglia region *n* = 11, brainstem *n* = 14). MBI, modified Barthel Index; NIHSS, National Institutes of Health Stroke Scale; BMI, body mass index; SD, standard deviation; Q1, first quartile; Q3, third quartile. *p* values are descriptive and nominal.

**Table 2:** Unadjusted respiratory outcomes by lesion-location group.

Outcome	Frontal Lobe	Basal Ganglia Region	Brainstem	*p* Value	Holm-Adjusted *p*
PIF, L/s	1.75 ± 0.73	2.28 ± 0.92	1.49 ± 0.60	0.00118	0.008
S-Index, cmH_2_O	33.75 ± 12.51	41.23 ± 15.62	31.69 ± 10.83	0.02498	0.150
VC, L	1.89 ± 0.70	1.95 ± 0.70	1.77 ± 0.73	0.65781	1.000
FVC, L	1.62 ± 0.66	1.83 ± 0.73	1.56 ± 0.72	0.34778	1.000
FEV1, L	1.01 ± 0.24	1.33 ± 0.60	0.97 ± 0.44	0.03600 *	0.180
PEF, L/s	1.57 ± 1.28	2.23 ± 1.36	1.72 ± 1.22	0.15115	0.605
MVV, L/min	23.49 ± 10.84	27.58 ± 13.15	23.31 ± 12.09	0.35259	1.000

* Welch ANOVA was used for FEV1 because of unequal variances; other outcomes used one-way ANOVA. Holm adjustment was calculated from unrounded *p* values for the seven omnibus tests, before rounding for presentation (Supplementary Table S7). PIF, peak inspiratory flow; VC, vital capacity; FVC, forced vital capacity; FEV1, forced expiratory volume in one second; PEF, peak expiratory flow; MVV, maximum voluntary ventilation; S-Index, strength index; ANOVA, analysis of variance; SD, standard deviation.

**Table 3:** Multivariable association of lesion location with respiratory outcomes.

Outcome	Overall *p*	Holm-Adjusted Overall *p*	Frontal vs. Brainstem β (95% CI); *p*	Basal Ganglia Region vs. Brainstem β (95% CI); *p*
PIF, L/s	0.00126	0.009	0.260 (−0.031 to 0.552); 0.080	0.625 (0.282 to 0.967); <0.001
S-Index, cmH_2_O	0.06447	0.322	2.02 (−2.96 to 7.00); 0.428	6.36 (1.00 to 11.72); 0.020
VC, L	0.69233	1.000	0.120 (−0.167 to 0.406); 0.412	0.075 (−0.182 to 0.332); 0.569
FVC, L	0.35381	1.000	0.056 (−0.271 to 0.384); 0.737	0.220 (−0.103 to 0.544); 0.182
FEV1, L	0.04389	0.263	0.062 (−0.133 to 0.257); 0.531	0.308 (0.044 to 0.571); 0.022
PEF, L/s	0.38844	1.000	−0.118 (−0.816 to 0.580); 0.740	0.377 (−0.338 to 1.092); 0.301
MVV, L/min	0.79002	1.000	1.18 (−4.17 to 6.53); 0.665	2.07 (−4.00 to 8.14); 0.504

Brainstem is the reference group. Models are adjusted for age, sex, height, smoking history, coronary heart disease, and stroke type and used HC3 robust standard errors. β represents the adjusted absolute difference; CI, confidence interval; HC3, type 3 heteroscedasticity-consistent covariance estimator; PIF, peak inspiratory flow; S-Index, strength index; VC, vital capacity; FVC, forced vital capacity; FEV1, forced expiratory volume in one second; PEF, peak expiratory flow; MVV, maximum voluntary ventilation. Holm-adjusted overall *p* values were calculated from unrounded omnibus *p* values across the seven adjusted outcome models, before rounding for presentation. Supplementary Table S7 provides the numerical audit.

**Table 4:** Sensitivity analysis restricted to ischemic stroke (*n* = 59).

Outcome	Overall *p*	Frontal vs. Brainstem β (95% CI); *p*	Basal Ganglia Region vs. Brainstem β (95% CI); *p*
PIF, L/s	<0.001	0.315 (−0.048 to 0.678); 0.089	0.792 (0.400 to 1.185); <0.001
S-Index, cmH_2_O	0.018	1.97 (−4.32 to 8.26); 0.540	8.92 (2.73 to 15.12); 0.005
FEV1, L	0.155	−0.011 (−0.281 to 0.260); 0.938	0.250 (−0.072 to 0.571); 0.128

Brainstem is the reference group. Models adjusted for age, sex, height, smoking history, and coronary heart disease and used HC3 robust standard errors. This analysis was treated as a sensitivity analysis; *p* values are nominal. CI, confidence interval; HC3, type 3 heteroscedasticity-consistent covariance estimator; PIF, peak inspiratory flow; S-Index, strength index; FEV1, forced expiratory volume in one second.

**Table 5:** Additional PIF analyses with clinical adjustment and restriction to first-ever stroke.

Sample and Model	Frontal vs. Brainstem β (95% CI); *p*	Basal Ganglia Region vs. Brainstem β (95% CI); *p*	Overall *p*	Adjusted R^2^
Full cohort (*n* = 80), M0	0.260 (−0.031 to 0.552); 0.080	0.625 (0.282 to 0.967); <0.001	0.00126	0.463
Full cohort (*n* = 80), M1	0.256 (−0.046 to 0.558); 0.097	0.614 (0.269 to 0.959); <0.001	0.002	0.457
Full cohort (*n* = 80), M2	0.275 (−0.010 to 0.560); 0.059	0.636 (0.300 to 0.973); <0.001	<0.001	0.550
Full cohort (*n* = 80), M3	0.200 (−0.079 to 0.479); 0.160	0.541 (0.200 to 0.882); 0.002	0.008	0.535
Ischemic stroke (*n* = 59), M0	0.315 (−0.048 to 0.678); 0.089	0.792 (0.400 to 1.185); <0.001	<0.001	0.415
Ischemic stroke (*n* = 59), M1	0.314 (−0.063 to 0.691); 0.103	0.791 (0.390 to 1.192); <0.001	<0.001	0.403
Ischemic stroke (*n* = 59), M2	0.296 (−0.049 to 0.641); 0.093	0.721 (0.331 to 1.111); <0.001	0.001	0.515
Ischemic stroke (*n* = 59), M3	0.216 (−0.139 to 0.572); 0.234	0.650 (0.232 to 1.068); 0.002	0.010	0.472
First-ever stroke (*n* = 63), M0	0.080 (−0.305 to 0.465); 0.684	0.507 (0.064 to 0.950); 0.025	0.073	0.474
First-ever stroke (*n* = 63), M1	0.067 (−0.335 to 0.470); 0.742	0.489 (0.045 to 0.932); 0.031	0.086	0.473
First-ever stroke (*n* = 63), M2	0.112 (−0.271 to 0.495); 0.566	0.492 (0.052 to 0.932); 0.028	0.079	0.564
First-ever stroke (*n* = 63), M3	0.019 (−0.355 to 0.393); 0.919	0.381 (−0.067 to 0.829); 0.096	0.197	0.544

PIF, peak inspiratory flow; CI, confidence interval; NIHSS, National Institutes of Health Stroke Scale; MBI, modified Barthel Index; R^2^, coefficient of determination. PIF differences are in L/s. M0 is the original six-covariate model; M1 adds time since stroke; M2 adds NIHSS to M1; M3 adds MBI to M1. Ischemic-stroke models omit stroke type. Brainstem is the reference category. All *p* values in this table are nominal; these analyses do not constitute an additional Holm-corrected outcome family.

## Data Availability

The original contributions presented in this study are included in the article and Supplementary Materials. Further inquiries can be directed to the corresponding author.

## Supplementary Materials

The following supporting information can be downloaded at: https://www.mdpi.com/article/10.3390/arm94050076/s1. Supplementary File S1 contains the STROBE reporting checklist; Supplementary Table S1: Initial imaging classification agreement; Supplementary Table S2: PIF model fit and multicollinearity; Supplementary Table S3: Original-outcome model diagnostics; Supplementary Table S4: PIF influence and omission checks; Supplementary Table S5: PIF analyses restricted to participants with a smoking history; Supplementary Table S6: Full cohort PIF sensitivity analysis using smoking pack years; Supplementary Table S7: Omnibus *p* values and Holm adjustment audit; Supplementary Figure S1: Original PIF model diagnostics. Full cohort: (a) residual and influence plots; (b) partial residual plots; Supplementary Figure S2: Original PIF model diagnostics. Ischemic stroke: (a) residual and influence plots; (b) partial residual plots. STROBE, Strengthening the Reporting of Observational Studies in Epidemiology; PIF, peak inspiratory flow.

## Abbreviations

ANOVA	analysis of variance
BMI	body mass index
CI	confidence interval
FEV1	forced expiratory volume in one second
FVC	forced vital capacity
HC3	heteroscedasticity-consistent covariance estimator, type 3
MBI	modified Barthel Index
NIHSS	National Institutes of Health Stroke Scale
VIF	variance inflation factor
MVV	maximum voluntary ventilation
PEF	peak expiratory flow
PIF	peak inspiratory flow
S-Index	strength index
STROBE	Strengthening the Reporting of Observational Studies in Epidemiology
VC	vital capacity
ATS	American Thoracic Society
COPD	chronic obstructive pulmonary disease
CT	computed tomography
ERS	European Respiratory Society
MRI	magnetic resonance imaging
mRS	modified Rankin Scale
Q1	first quartile
Q3	third quartile
SD	standard deviation

## References

[1] Drakopanagiotakis F., Bonelis K., Steiropoulos P., Tsiptsios D., Sousanidou A., Christidi F., Gkantzios A., Serdari A., Voutidou S., Takou C.-M. (2024). Pulmonary function tests post-stroke: Correlation between lung function, severity of stroke, and improvement after respiratory muscle training. Neurol. Int..

[2] Yildiz A., Mustafaoglu R., Bardak A.N. (2024). Respiratory muscle strength in stroke: A case-control study. Rev. Assoc. Med. Bras..

[3] Chen Y., Zhou S., Liao L., He J., Tang D., Wu W., Wang K. (2023). Diaphragmatic ultrasound can help evaluate pulmonary dysfunction in patients with stroke. Front. Neurol..

[4] Li M., Huang Y., Chen H., Wang S., Zhou Y., Zhang Y. (2024). Relationship between motor dysfunction, the respiratory muscles and pulmonary function in stroke patients with hemiplegia: A retrospective study. BMC Geriatr..

[5] Khedr E.M., El Shinawy O., Khedr T., Abdel Aziz Ali Y., Awad E.M. (2000). Assessment of corticodiaphragmatic pathway and pulmonary function in acute ischemic stroke patients. Eur. J. Neurol..

[6] Herrero J.L., Khuvis S., Yeagle E., Cerf M., Mehta A.D. (2018). Breathing above the brain stem: Volitional control and attentional modulation in humans. J. Neurophysiol..

[7] McKay L.C., Adams L., Frackowiak R.S.J., Corfield D.R. (2008). A bilateral cortico-bulbar network associated with breath holding in humans, determined by functional magnetic resonance imaging. NeuroImage.

[8] Koritnik B., Azam S., Andrew C.M., Leigh P.N., Williams S.C.R. (2009). Imaging the brain during sniffing: A pilot fMRI study. Pulm. Pharmacol. Ther..

[9] Lee S.J., Lee K.W., Kim S.B., Lee J.H., Park M.K. (2015). Voluntary cough and swallowing function characteristics of acute stroke patients based on lesion type. Arch. Phys. Med. Rehabil..

[10] Lee K.B., Lim S.H., Park G.Y., Im S. (2020). Effect of brain lesions on voluntary cough in patients with supratentorial stroke: An observational study. Brain Sci..

[11] Lee K.B., Kim M.K., Jeong J.R., Lee W.H. (2016). Reliability of an electronic inspiratory loading device for assessing pulmonary function in post-stroke patients. Med. Sci. Monit..

[12] Silva P.E., de Carvalho K.L., Frazão M., Maldaner V., Daniel C.R., Gomes-Neto M. (2018). Assessment of maximum dynamic inspiratory pressure. Respir. Care.

[13] Minahan C., Sheehan B., Doutreband R., Kirkwood T., Reeves D., Cross T. (2015). Repeated-sprint cycling does not induce respiratory muscle fatigue in active adults: Measurements from the POWERbreathe inspiratory muscle trainer. J. Sports Sci. Med..

[14] Souza V.C., Saldanha M.F.L.S., Ferreira E.V.M., Nery L.E., Sperandio P.C.A. (2025). Maximal dynamic inspiratory pressure: S-Index prediction values and diagnosis accuracy. J. Bras. Pneumol..

[15] Kuo H.-C., Shi M.F., Liu G.H., Liu Y.Y., Liu B.Z. (2024). Effects of conventional respiratory training combined with articulatory visual feedback training on respiratory function in stroke patients. Fudan Univ. J. Med. Sci..

[16] von Elm E., Altman D.G., Egger M., Pocock S.J., Gøtzsche P.C., Vandenbroucke J.P., STROBE Initiative (2007). The Strengthening the Reporting of Observational Studies in Epidemiology (STROBE) statement: Guidelines for reporting observational studies. Lancet.

[17] Areias G.S., Santiago L.R., Teixeira D.S., Reis M.S. (2020). Concurrent validity of the static and dynamic measures of inspiratory muscle strength: Comparison between maximal inspiratory pressure and S-Index. Braz. J. Cardiovasc. Surg..

[18] Graham B.L., Steenbruggen I., Miller M.R., Barjaktarevic I.Z., Cooper B.G., Hall G.L., Hallstrand T.S., Kaminsky D.A., McCarthy K., McCormack M.C. (2019). Standardization of spirometry 2019 update: An official American Thoracic Society and European Respiratory Society technical statement. Am. J. Respir. Crit. Care Med..

[19] Stanojevic S., Kaminsky D.A., Miller M.R., Thompson B., Aliverti A., Barjaktarevic I., Cooper B.G., Culver B., Derom E., Hall G.L. (2022). ERS/ATS technical standard on interpretive strategies for routine lung function tests. Eur. Respir. J..

[20] Long J.S., Ervin L.H. (2000). Using heteroscedasticity consistent standard errors in the linear regression model. Am. Stat..

[21] Cook R.D. (1977). Detection of influential observation in linear regression. Technometrics.

[22] Liu F., Jones A.Y.M., Tsang R.C.C., Yam T.T.T., Tsang W.W.N. (2025). Diaphragm and sternocleidomastoid muscle activity with increasing inspiratory pressure loads in people after stroke. Sci. Rep..

[23] Boz K., Saka S., Çetinkaya İ. (2023). The relationship of respiratory functions and respiratory muscle strength with trunk control, functional capacity, and functional independence in post-stroke hemiplegic patients. Physiother. Res. Int..

