# Supplementary material for: Stroke Lesion Location Is Associated with Dynamic Inspiratory Performance: A Cross-Sectional Study
Source: Adv Respir Med. 2026 Oct 20;94(5):76. doi: 10.3390/arm94050076 (PMC13701287; doi:10.3390/arm94050076)
Supplement: Supplementary file 1 [file arm-94-00076-s001.zip › arm-4583378-supplementary.pdf]

Supplementary File S1

Stroke Lesion Location Is Associated with Dynamic Inspiratory Performance: A Cross-Sectional Study

Supplementary File S1 contains the STROBE reporting checklist, Supplementary Tables S1–S7, and Supplementary Figures S1 and S2. The tables present initial imaging agreement, PIF model fit and multicollinearity, original-outcome model diagnostics, PIF influence checks, smoking-exposure sensitivity analyses, and the omnibus *p*-value and Holm-adjustment audit. PIF, peak inspiratory flow; STROBE, Strengthening the Reporting of Observational Studies in Epidemiology.

STROBE reporting checklist

Item	Reporting topic	Manuscript location or explanation
1a	Design in title	Title explicitly identifies a cross-sectional study.
1b	Abstract	Design, sources, outcomes, estimates, and limitations are summarized.
2	Background	Introduction, paragraphs 1–3.
3	Objectives	Introduction, final paragraph.
4	Study design	Section 2.1, paragraph 1.
5	Setting and dates	Section 2.1, paragraph 1; Figure 1. Two source periods are distinguished.
6a	Participant selection	Sections 2.1–2.2; Results, opening paragraph; Figure 1. Eligibility criteria apply to both sources. Of 30 earlier enrolled trial participants, 20 were included; of 90 subsequent consecutively screened patients, 60 were included. The source trial's original screening denominator is not represented.
7	Variables	Sections 2.1–2.5; broad lesion definitions and respiratory outcomes are specified.
8	Measurement comparability	Sections 2.1–2.4. Same respiratory assessor and procedure; source-cohort comparability limitations in Discussion.
9	Bias	Imaging and respiratory blinding in Sections 2.2–2.3; selection, source, measurement, and confounding limitations in Discussion.
10	Study size	Section 2.5 and Figure 1. No anticipated lesion-effect sample-size calculation.
11	Quantitative variables	Sections 2.3–2.5; Table 1 notes and Tables 2–5.
12a	Statistical methods	Section 2.5. SPSS version 26.0 and Python version 3.14; covariates, HC3 inference, and multiplicity correction. Supplementary Table S7 reports the audit using unrounded omnibus p values.
12b	Subgroups	Section 2.5; Tables 4–5. Ischemic and first-ever-stroke restrictions.
12c	Missing data	Sections 2.3–2.5; Results, opening paragraphs; Figure 1. Two earlier participants lacked baseline data required for this paper because of withdrawal and were not analyzed. Six subsequent patients did not complete the prescribed assessments. These non-inclusions are distinguished from completeness of model variables in the final 80-person sample and from unavailable maneuver-level quality records.
12d	Sampling strategy	Section 2.1. Later recruitment was consecutive; no complex sampling or survey weights.
12e	Sensitivity analyses	Section 2.5; Table 5; Supplementary Tables S2–S6.
13a	Participant counts	Results, opening paragraph; Figure 1. Total assessed for inclusion: 120. Earlier enrolled trial participants: 30, with 20 included and 10 not included. Subsequent screened patients: 90, with 60 included and 30 not included. Final analytical sample: 80 (20 + 60); frontal lobe 27, basal ganglia region 26, brainstem 27.

Item	Reporting topic	Manuscript location or explanation
13b	Non-participation reasons	Section 2.1; Results, opening paragraph; Figure 1. Earlier non-inclusion: lesions involving multiple predefined anatomical categories, 8; required baseline data unavailable because of withdrawal, 2. Subsequent non-inclusion: eligibility criteria not met, 22; prescribed assessments not completed, 6; consent withdrawn after informed consent and before study assessments, 2. Total 40; one reason per person. Earlier lesion-based non-inclusion does not imply withdrawal from the source trial; later consent withdrawal is not loss to longitudinal follow-up.
13c	Flow diagram	Figure 1 in Section 2.1 shows both recruitment periods, stage-specific non-inclusion reasons, 20 + 60 = 80 analyzed participants, and anatomical group counts of 27/26/27. The caption distinguishes the earlier enrolled trial cohort from the subsequent screening population.
14a	Participant characteristics	Table 1, including overall and group timing, NIHSS, MBI, and smoking pack-years.
14b	Variable completeness	Results and Table 1 notes; Sections 2.3–2.5. Model variables are complete in the final 80-person analytical sample; zero pack-years in participants without a smoking history are not imputed. This completeness statement does not include the two earlier participants with unavailable required baseline data or the six subsequent assessment non-completers.
15	Outcome summaries	Table 2 and Results FEV1/FVC paragraph.
16a	Effect estimates	Tables 3–5, with covariates and uncertainty specified.
16b	Category boundaries	Section 2.2. Broad anatomical categories; adjacent internal capsule and brainstem levels.
16c	Absolute effects	PIF contrasts reported directly in L/s; no risk translation applicable.
17	Other analyses	Tables 4–5; Supplementary Tables S1–S7 and Figures S1–S2.
18	Key results	Discussion, opening paragraph.
19	Limitations	Section 4, final three paragraphs. Precision, confounding, selection, source periods, anatomy, and measurement limitations.
20	Interpretation	Discussion and Conclusions; first-ever-stroke weakening and exploratory status retained.
21	Generalizability	Discussion, selection and anatomy limitations; observed timing range.
22	Funding	Funding statement: no external funding.

Reporting topics are abbreviated for this manuscript. Item identifiers follow the STROBE cross-sectional checklist. Source: Strengthening the Reporting of Observational Studies in Epidemiology (STROBE) Initiative, https://www.strobe-statement.org/checklists/; see Reference 16 in the main manuscript.

Supplementary Table S1 Initial imaging classification agreement

Physician A / Physician B	Frontal lobe	Basal ganglia region	Brainstem	Total
Frontal lobe	25	2	0	27
Basal ganglia region	1	24	1	26
Brainstem	1	1	25	27
Total	27	27	26	80

These are the actual independent classifications before consensus. Agreement was 74/80 (92.5%) and disagreement 6/80 (7.5%). Unweighted Cohen's κ = 0.888 (95% CI, 0.801–0.974); the asymptotic standard error was 0.0442. The three categories have no ordinal scale. CI, confidence interval.

Supplementary Table S2 PIF model fit and multicollinearity

Sample and model	*n*	k	R^2	Adjusted R^2	Lesion partial R^2	Maximum VIF
Full cohort, M0	80	9	0.518	0.463	0.161	2.010
Full cohort, M1	80	10	0.519	0.457	0.154	2.119
Full cohort, M2	80	11	0.607	0.550	0.193	2.119
Full cohort, M3	80	11	0.593	0.535	0.142	2.221
Ischemic stroke, M0	59	8	0.486	0.415	0.226	1.904
Ischemic stroke, M1	59	9	0.486	0.403	0.222	2.193
Ischemic stroke, M2	59	10	0.591	0.515	0.227	2.193
Ischemic stroke, M3	59	10	0.554	0.472	0.177	2.220

M0: original covariates (age, sex, height, smoking history, coronary heart disease, stroke type). M1: M0 plus time since stroke. M2: M1 plus NIHSS. M3: M1 plus MBI. Ischemic models omit stroke type. k includes the intercept and both lesion terms. Partial R^2 is (SSE of covariate-only model − SSE of full model) / SSE of covariate-only model. It is conditional explanatory contribution, not a causal share of total variance. PIF, peak inspiratory flow; NIHSS, National Institutes of Health Stroke Scale; MBI, modified Barthel Index; SSE, sum of squared errors; VIF, variance inflation factor; R^2, coefficient of determination.

Supplementary Table S3 Original-outcome model diagnostics

Sample and outcome	Adjusted R^2	Maximum VIF	Shapiro–Wilk *p*	Breusch–Pagan *p*	Fitted-power screen *p*	Cook flags
Full cohort<br>PIF	0.463	2.010	0.676	0.070	0.259	3
Full cohort<br>S-Index	0.467	2.010	0.528	0.240	0.006	3
Full cohort<br>VC	0.538	2.010	0.641	0.413	0.275	7
Full cohort<br>FVC	0.395	2.010	0.419	0.093	0.006	3
Full cohort<br>FEV1	0.321	2.010	0.065	0.151	0.002	3
Full cohort<br>PEF	0.066	2.010	<0.001	0.132	0.274	5
Full cohort<br>MVV	0.280	2.010	0.004	0.214	0.037	5
Ischemic stroke<br>PIF	0.415	1.904	0.755	0.078	0.318	2
Ischemic stroke<br>S-Index	0.420	1.904	0.708	0.249	0.522	4
Ischemic stroke<br>VC	0.479	1.904	0.244	0.442	0.391	3
Ischemic stroke<br>FVC	0.347	1.904	0.414	0.162	0.013	3
Ischemic stroke<br>FEV1	0.190	1.904	0.074	0.161	0.055	5
Ischemic stroke<br>PEF	0.037	1.904	<0.001	0.244	0.369	4
Ischemic stroke<br>MVV	0.138	1.904	0.003	0.694	0.170	5

All *p* values are nominal diagnostic screens. The functional-form screen jointly tests added squared and cubed standardized fitted values using HC3 Wald inference; it does not test every possible source of misspecification. Cook flags use $D > 4/n$. PEF and MVV residual screens and some secondary-outcome fitted-power screens raised concerns. Non-significant screens do not prove assumptions, and HC3 does not correct functional form or residual confounding. No primary model was replaced solely on these screens. HC3, type 3 heteroscedasticity-consistent covariance estimator; PIF, peak inspiratory flow; S-Index, strength index; VC, vital capacity; FVC, forced vital capacity; FEV1, forced expiratory volume in one second; PEF, peak expiratory flow; MVV, maximum voluntary ventilation; VIF, variance inflation factor; R^2, coefficient of determination.

Supplementary Table S4 PIF influence and omission checks

Sample and model	Cook flags / *n* retained	Leave-one-out basal β range	Joint-omission basal β (95% CI)	Joint-omission frontal β (95% CI)	Largest leave-one-out overall *p*
Full cohort M0	3 / 77	0.574 to 0.677	0.551 (0.232 to 0.869)	0.299 (0.018 to 0.581)	0.003
Full cohort M1	4 / 76	0.567 to 0.662	0.536 (0.208 to 0.863)	0.253 (−0.035 to 0.541)	0.004
Full cohort M2	5 / 75	0.596 to 0.705	0.687 (0.375 to 0.999)	0.312 (0.054 to 0.571)	0.002
Full cohort M3	5 / 75	0.488 to 0.602	0.546 (0.238 to 0.854)	0.194 (−0.060 to 0.448)	0.016
Ischemic stroke M0	2 / 57	0.722 to 0.866	0.667 (0.303 to 1.032)	0.315 (−0.033 to 0.663)	<0.001
Ischemic stroke M1	3 / 56	0.724 to 0.857	0.792 (0.408 to 1.177)	0.285 (−0.088 to 0.658)	0.001
Ischemic stroke M2	5 / 54	0.667 to 0.800	0.746 (0.392 to 1.101)	0.323 (0.023 to 0.624)	0.003
Ischemic stroke M3	4 / 55	0.559 to 0.716	0.630 (0.256 to 1.003)	0.248 (−0.072 to 0.568)	0.021

PIF estimates are in L/s, with brainstem as reference. Joint omission removes all observations flagged by Cook's D >4/*n* in that model, for exploration only; main models retain every participant. In all eight full/ischemic PIF models, leave-one-out basal contrasts retained positive CIs excluding zero. The original maximum Cook's D was 0.107 (full cohort) and 0.149 (ischemic). Some joint-omission frontal CIs excluded zero, unlike the main-model frontal contrasts. These changes are reported without upgrading them to main findings. First-ever-stroke analyses in Table 5 are not overridden by these omission checks; their overall tests were nonsignificant. PIF, peak inspiratory flow; CI, confidence interval; β, adjusted difference.

Supplementary Table S5 PIF analyses restricted to participants with a smoking history

Model (*n* = 42)	Frontal vs brainstem β (95% CI); *p*	Basal ganglia region vs brainstem β (95% CI); *p*	Overall lesion *p*
Original remaining covariates	0.211 (−0.202 to 0.624); 0.316	0.725 (0.198 to 1.251); 0.007	0.025
Additional pack-year adjustment	0.273 (−0.160 to 0.705); 0.217	0.754 (0.200 to 1.308); 0.008	0.028

PIF contrasts are in L/s. Both models retain age, sex, height, coronary heart disease, and stroke type. Smoking history is constant in this subset and is omitted. The second model additionally includes continuous pack-years. Estimates use HC3 standard errors, normal 95% CIs and coefficient tests, and a two-degree-of-freedom Wald χ^2 test for the lesion terms. All *p* values are nominal and exploratory. PIF, peak inspiratory flow; HC3, type 3 heteroscedasticity-consistent covariance estimator; CI, confidence interval; β, adjusted difference.

All 42 participants with a smoking history had recorded pack-years: frontal lobe *n* = 17, basal ganglia region *n* = 11, and brainstem *n* = 14. This table remains an exploratory subset analysis. The 38 participants with documented absence of smoking history had blank source entries denoting zero exposure; the full-cohort analysis applying that definition is reported separately in Supplementary Table S6. Directional consistency does not rule out residual confounding.

Before and after adding pack-years, the models included 8 and 9 parameters including the intercept, adjusted R^2 was 0.607 and 0.617, maximum VIF was 2.11 and 2.15, and lesion-category partial R^2 was 0.229 and 0.245. These are conditional statistical fit measures, not causal explanatory fractions.

Cook's D >4/42 flagged four observations in each model; maximum Cook's D was 0.187 and 0.250. Main estimates retained all 42 participants. In the pack-year-adjusted model, leave-one-out basal ganglia region–brainstem estimates ranged from 0.639 to 0.907 L/s and their CIs excluded zero, whereas the overall lesion-category *p* ranged from 0.003 to 0.056. Joint omission of the four flagged observations yielded 0.771 L/s (95% CI, 0.320–1.222; *n* = 38; overall *p* = 0.004). Thus, the contrast retained its direction, but nominal overall significance was sensitive to omission of individual observations. These influence checks are exploratory and do not constitute comprehensive validation of this small subset model.

Supplementary Table S6 Full cohort PIF sensitivity analysis using smoking pack years

Both models include the two lesion-category terms, age, sex, height, coronary heart disease, and stroke type. The original model uses binary smoking history; the sensitivity model replaces it with continuous pack-years. The 38 participants with documented absence of smoking history contribute 0 pack-years; these zeros are not imputed unknown exposures. This revision-stage analysis is exploratory and retains all 80 participants. Estimates use HC3 standard errors, normal 95% CIs, and a two-degree-of-freedom Wald χ^2 test. All *p* values here are nominal; the original seven-outcome Holm family is unchanged. The original-model omnibus *p* value is 0.00125777205930; its Holm-adjusted *p* across the original seven outcomes is 0.00880440441510, reported as 0.009 in the manuscript. The replacement-model *p* value is nominal and is not substituted into that Holm family. PIF, peak inspiratory flow; HC3, type 3 heteroscedasticity-consistent covariance estimator; CI, confidence interval; β, adjusted difference.

**Model *n* = 80**	**Frontal lobe vs brainstem β (95% CI)**	**Basal ganglia region vs brainstem β (95% CI)**	**Overall *p***
Original binary smoking history	0.260 (−0.031–0.552)	0.625 (0.282–0.967)	0.00125777205930
Continuous pack years replacement	0.270 (−0.018–0.559)	0.624 (0.281–0.966)	0.00116872013150

PIF differences are in L/s. Original and replacement models each contain nine parameters including the intercept. Adjusted R^2 was 0.463 and 0.467, respectively; maximum VIF was 2.01 and 1.99. In the replacement model, leave-one-out overall *p* values ranged from 0.00034 to 0.00253, and all basal ganglia region–brainstem contrast CIs remained above zero. The screening thresholds did not determine exclusions: all 80 participants were retained. These limited fit and influence checks do not establish that all model assumptions were met or that smoking-related confounding was eliminated.

Supplementary Table S7 Omnibus *p* values and Holm adjustment audit

Both families contain the same seven original respiratory outcomes in the full analytical cohort (*n* = 80). Adjusted omnibus tests are two-degree-of-freedom HC3 Wald χ^2 tests from the original six-covariate models. Holm adjustment was computed from full-precision *p* values before reporting; additional digits are shown here for audit. The PIF unadjusted and adjusted Holm values are reported as 0.008 and 0.009, respectively. S-Index and FEV1 adjusted Holm values are 0.322 and 0.263. The full-cohort pack-year replacement model remains a separate exploratory analysis with a nominal *p* value. FEV1/FVC does not expand the original outcome family. HC3, type 3 heteroscedasticity-consistent covariance estimator; PIF, peak inspiratory flow; S-Index, strength index; VC, vital capacity; FVC, forced vital capacity; FEV1, forced expiratory volume in one second; PEF, peak expiratory flow; MVV, maximum voluntary ventilation.

Outcome	Unadjusted *p*	Unadjusted Holm *p*	Adjusted model *p*	Adjusted model Holm *p*
PIF	0.00118478005745	0.00829346040214	0.0012577720593	0.0088044044151
S-Index	0.0249846339917	0.14990780395	0.0644703877545	0.322351938772
VC	0.657812212003	1	0.692326162841	1
FVC	0.347778881601	1	0.35380776405	1
FEV1	0.0360026038871	0.180013019435	0.0438913469486	0.263348081692
PEF	0.151145510341	0.604582041363	0.388440563236	1
MVV	0.352587814881	1	0.790016268198	1

Supplementary Figure S1 Original PIF model diagnostics

Full cohort: (a) residual and influence plots; (b) partial residual plots.

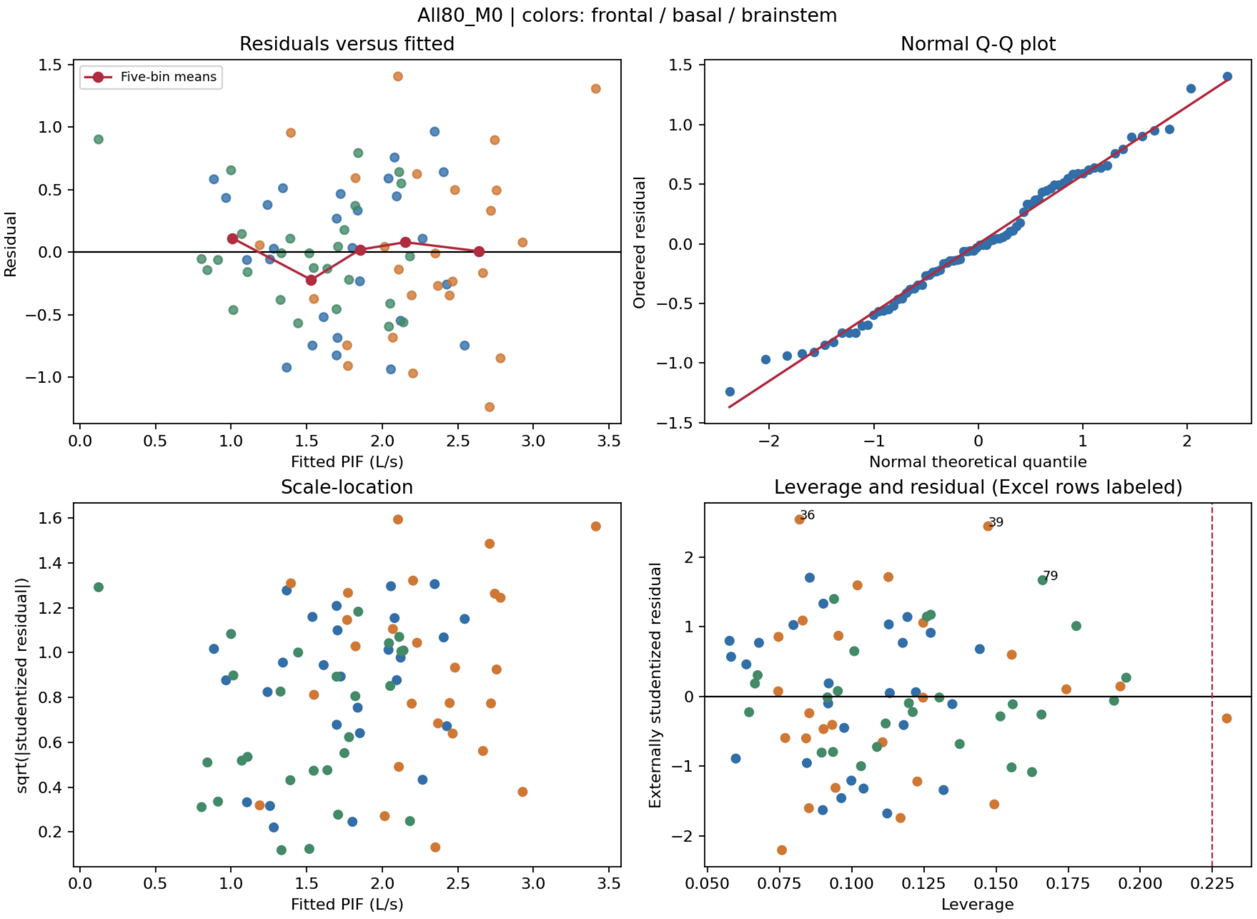

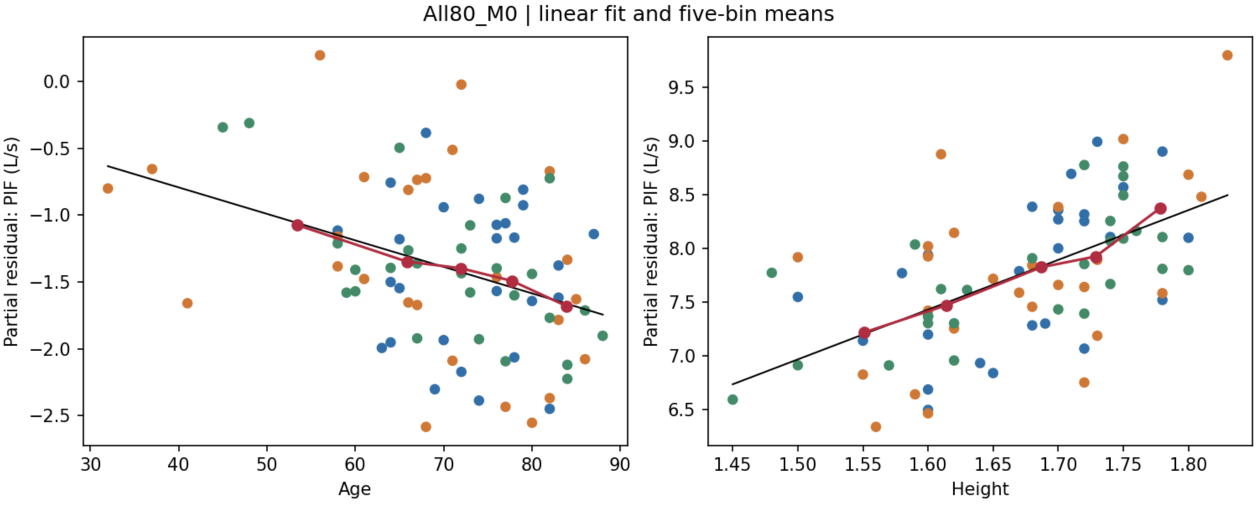

Colors identify frontal, basal ganglia region, and brainstem categories. Labeled numbers refer to source spreadsheet row positions, not patient names. Red binned means are descriptive; the vertical dashed line is the leverage screening threshold. The plots supplement the numerical diagnostics and do not demonstrate absence of confounding.

Supplementary Figure S2 Original PIF model diagnostics

Ischemic stroke: (a) residual and influence plots; (b) partial residual plots.

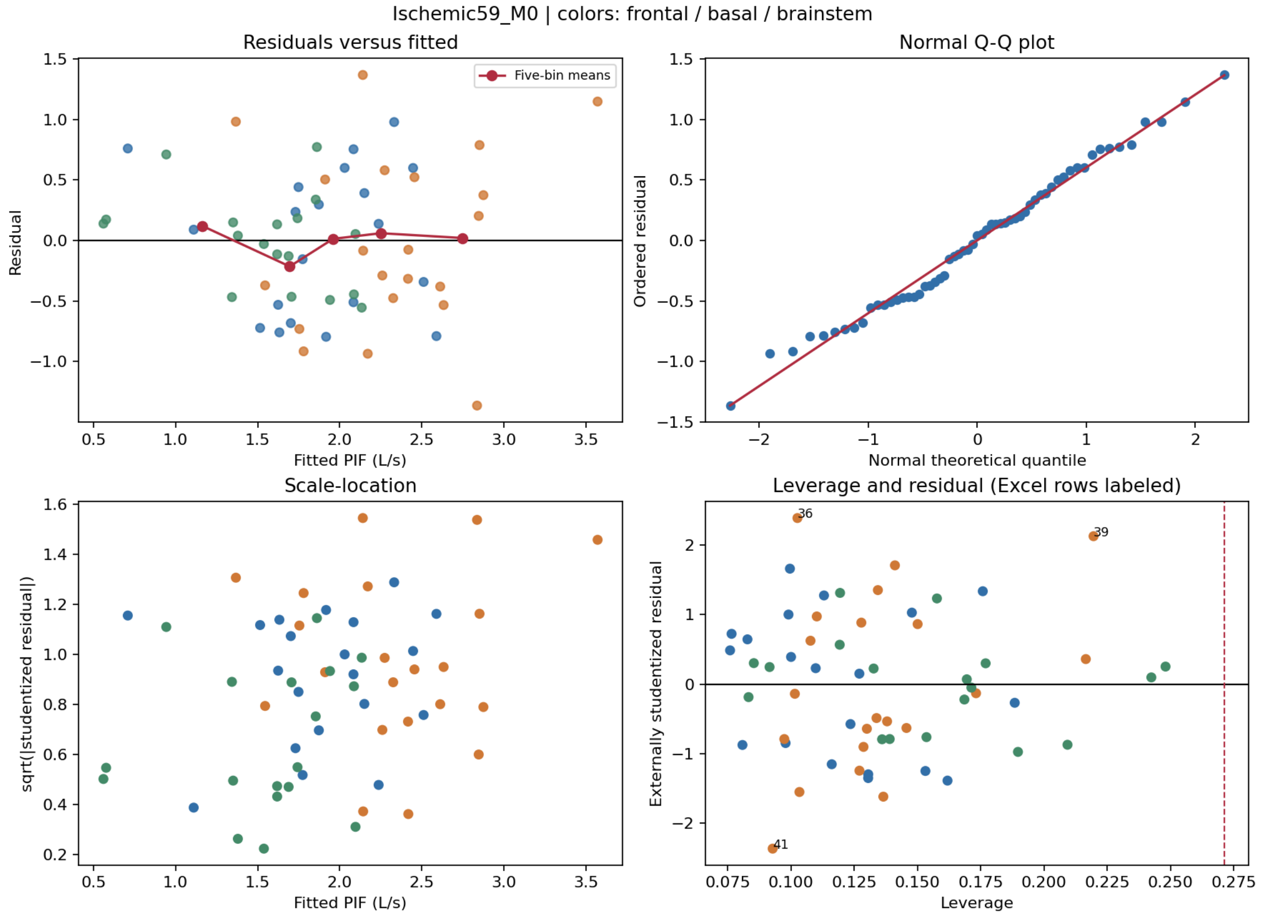

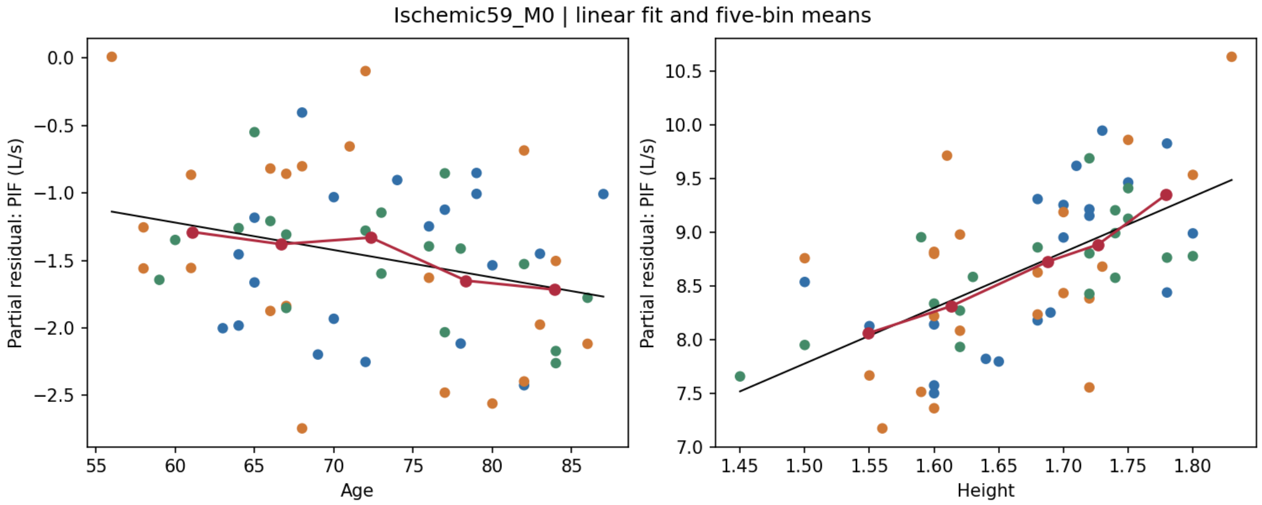

Colors identify frontal, basal ganglia region, and brainstem categories. Labeled numbers refer to source spreadsheet row positions, not patient names. Red binned means are descriptive; the vertical dashed line is the leverage screening threshold. The plots supplement the numerical diagnostics and do not demonstrate absence of confounding.
